# Learn from the best hospitals: a comparison of the mission, vision and values

**DOI:** 10.1186/s12913-023-09699-8

**Published:** 2023-07-25

**Authors:** Xiaoping Qin, Bing-Long Wang, Jinhong Zhao, Peixin Wu, Tingfang Liu

**Affiliations:** 1 School of Public Health, The University of Sydney, Sydney, NSW Australia; 2 School of Health Policy and Management, Chinese Academy of Medical Sciences and Peking Union Medical College, Beijing, China; 3 Peking Union Medical College Hospital, Beijing, China

**Keywords:** Culture, Mission, Vision, Value, Best hospitals

## Abstract

**Background:**

The hospital’s mission, vision, and values are the core of the hospital’s culture and the most profound expression of the hospital’s culture. Although there have been many comparative studies on the mission, vision and values of organizations in the past, there have been few studies on the mission, vision and values of hospitals in the healthcare field. The purpose is to understand how the world’s top hospitals develop the use of mission, vision and values in their “day-to-day management” and this may help other hospitals to develop their mission, vision and value effectively.

**Methods:**

This paper collects and discusses the approaches of the world’s top five hospitals in mission, vision and value through a qualitative analysis method. Documents for the study were collected from the publicly available information of the five hospitals, including their websites, annual reports, and relevant academic literature published in English on Google Scholar, PubMed, Medline, and Web of Science.

**Results:**

These five hospitals have similarities and differences in the development of their missions, visions and values, which are worthy of study by other hospitals. The setting of a mission is a useful reflection of the hospital’s focus and direction showing the social responsibility and sustainability of the hospital. The development of a vision has a guiding role in the equity and development of patients and employees and can improve the efficiency and effectiveness of hospital management and ensure the quality of services. The elaboration of values can greatly help hospitals to develop strategic plans and improve daily management.

**Conclusion:**

The top five hospitals in the world have several common valuable cultures in their missions, visions, and values, regardless of the properties of the hospitals or their management models. In addition, each hospital also has some enlightening descriptions that reflect their particularities.

## Background

The hospital’s mission, vision, and values are the core of the hospital’sculture and the most profound expression of the hospital’s culture [1]. While many previous comparative studies have been conducted on organizations’ mission, vision, and values [2, 3]. There are few studies on hospitals’ mission, vision, and value in the healthcare industry. Organizations’ articulation and cultivation of widely shared ownership and commitment to the purpose (i.e., the mission, vision, values, and goals) have long been recognized as critical to effective strategic planning for organizational improvement [4–6]. An organization’s mission is “what can we get for others,“ and the vision is “what can we get for ourselves?“ The values are “How can we achieve these two goals?“ [7]. A clear definition and focus on mission, vision, and values can give hospitals unique development [8]. Almost every healthcare organization in the US has made its cultural aspirations clear through its mission, vision statements, and values [9]. A mission statement is a short statement that describes the purpose and the reason for the hospital’s existence. A vision statement consists of the critical characteristics that leadership wants for the hospital’s future. Moreover, the vision statement is market-based and should reflect the overall direction desired for the hospital. The values statement defines the hospital’s guiding philosophy, ideals, and planning principles [10].

The hospital’s mission, vision, and values can be oriented to the general organization’s stakeholders [11]. For example, improving the quality and operational performance of healthcare services, influencing the willingness of patients to seek care, and helping hospitals communicate to patients whether their care meets the patient’s considerations [12]. It can also help employees recognize and integrate into the hospital’s organizational culture and select the suitable workplace for them [9] such as practicing social responsibility in medical services [13]. In short, when a hospital has the proper mission, vision, and values, it can get the right people in the right place to do the right thing [14].

Currently, few studies are focusing on the content of the hospital’s mission, vision, and values. Only one on the correlation between hospital managers and the mission statements of the hospitals to which they belong. This study showed a difference between the most popular type of mission statement content and managers’ impressions of its role in organizational performance [15]; However, this study did not directly examine the content of the mission statement itself. Some studies have also discussed the hospital’s approach to mission, vision, and values and found that mission and vision shape employee behavior and foster high levels of commitment, which ultimately improves employee performance and contributes to operational success [16]. Some studies of small and medium-sized organizations indicated a correlation between organizational vision and performance [11, 17, 18]. However, it has not been explored to a great extent in the healthcare industry [11]. This study explores the hospital’s commonalities and enlightening characteristics in mission, vision, and values from the top five best hospitals in the world.

### Theoretical foundation

Creating an organization’s mission, vision, and values is grounded in the Strategic Adaptation Theory. According to Mick & Wyttenback, this theory proposes that external and internal forces influence the actions taken by the organization to guide their environment and performance. Strategic intent allows an organization to evolve with goals, objectives, and expectations of superior performance [19]. Strategic Adaption theory also addresses categories of relationships important to the organization such as instrumental, institutional, and altruistic. Instrumental involves the exchange of resources between organizations to meet current and future needs. Institutional ties involve consideration of norms and expectations that provide greater legitimacy and credibility to the organization [20]. Finally, altruism addresses those relationships and behaviors that embrace a higher belief or value beyond an economic driver and is adapted because it is the right thing to do [21]. This is particularly important in healthcare because of the not-for-profit mission of many healthcare organizations.

More recently, strategic management theory asserts that organizations change their operations and priorities based on changing market conditions or shifting environmental factors [22]. The theory includes defining strategic management as the process and method for determining the organization’s objectives, polices, services, and priorities and allocating resources (such as people and financial) to implement the strategies and plans. It will often determine how the organization will compete in the market [22].

## Methods

Content analysis is a form of qualitative analysis used to examine the content of written materials to gain insight into the significance of the social activities described [23, 24]. The analytical process unlocks the knowledge contained in documents to provide insight into social phenomena [25]. The analysis was iterative and repetitive, similar to a dialogue in which we asked questions about the text, discussed new insights, asked further questions, and reiterated them. Discussions, reflections, and questions among the research team ensured consensus [26].

This study was conducted on the top five hospitals in Newsweek magazine’s “Best Hospitals” list for 2021 [27]. The World’s Best Hospitals 2021 ranking lists the best hospitals in 25 countries. These countries include the USA, Germany, Japan, Korea, France, Italy, UK, Spain, Brazil, Canada, India, Australia, Mexico, Netherlands, Poland, Austria, Thailand, Switzerland, Sweden, Belgium, Finland, Norway, Denmark, Israel and Singapore. These countries were selected primarily on the basis of standard of living/life expectancy, population size, number of hospitals and data availability.

**Fig. 1:**
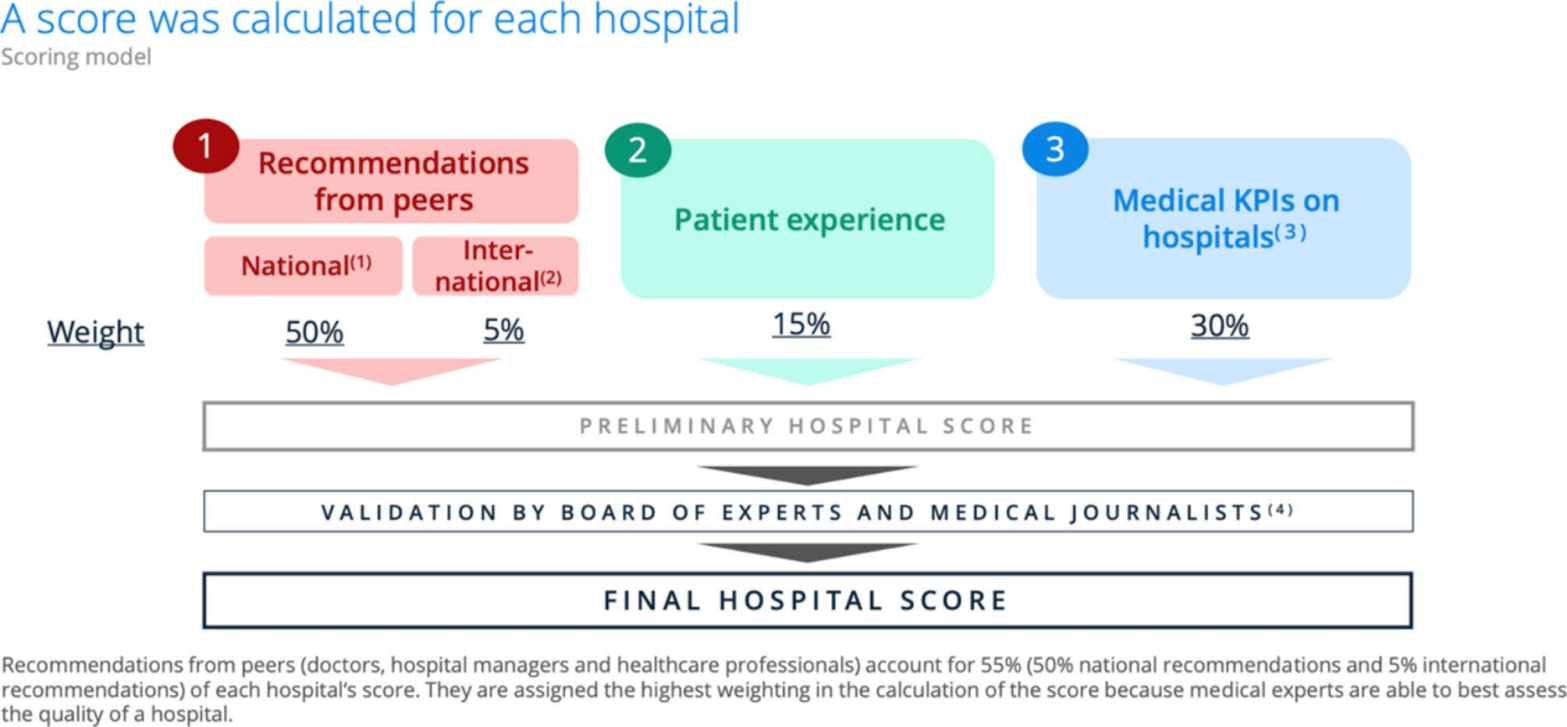
Newsweek magazine’s rating criteria for the best hospitals in the world [28]

In response to the methodology of the ranking, the magazine based its ranking on the following points (1) Recommendations from medical experts (doctors, hospital managers, medical professionals). (2) Results of patient surveys and patient follow-ups (3) Medical KPI indicators/clinical indicators on hospitals. The detailed scoring process is shown in Fig. 1 [28].

Each hospital in each country is given a score. Scores are only comparable between hospitals in the same country, as each country looks at different sources of patient experience and healthcare key performance indicators. As these data are not harmonized, it may be inaccurate to compare the performance of hospitals in different countries by using this score (for example, a score of 90 in country A does not necessarily mean that this hospital is better than a hospital with a score of 87 in country B). But the top five hospitals are all from North America, so are relatively less affected by this. However, the overall ranking of the journals is based on international recommendations from peers in different countries and incorporates the scores obtained by the hospitals. Therefore, the top five hospitals in the ranking are comparable [28].

The five hospitals in North America should have enlightening mission, vision, and values (MVV) in order to remain in the top 10 of the world’s best hospitals during the Covid-19 pandemic (2019–2021). Documentation for the study was collected from public information on the MVV of these five hospitals, including their websites, annual reports, and relevant academic literature published in English on Google Scholar, PubMed, Medline, and Web of Science. All documents were queried in 2021–2022, during which the MVV did not change. We conducted a precise inventory of the content of the documents through a document analysis method and extracted the characteristics of the content of the documents, and finally summarized the conclusions. We also invited five experts in the field of hospital management to give their expert opinions. The analysis was iterative, with the researchers asking questions about the content of the documents and having several discussions with the experts to avoid the personal subjective bias of the researchers. Information for this study was obtained from publicly available materials on the website.

### Data analysis

The data were imported for data analysis using the qualitative research software Nvivo 12, using the Content [29] analysis method: (1) reading the transcribed text of the interviews carefully and repeatedly until a sense of the whole emerges. (2) breaking down the material and analysing it line by line to identify significant statements and coding them. (3) coding and categorising recurring statements, things and phenomena to produce themes. (4) finding connections between themes to form clusters of themes. (5) repeating this cycle until saturation, i.e. no new themes and sub-themes are presented.

All the data were read carefully, compared and analysed for significant statements, similarities were identified, themes were identified, and the results summarised and compared with the original data to determine the accuracy of the themes. The information is discussed by multiple researchers to refine ideas [29, 30].

Table 1 shows the information we collected on the five hospitals’ characteristics, including Mayo Clinic(MC) [31], Cleveland Clinic(CC) [32], Massachusetts General Hospital(MGH) [33], Toronto General Hospital–University Health Network(UHN)[34], and Johns Hopkins Hospital(JHH)[35]. The five hospitals are all founded more than 100 years ago. MGH and CC were the first and the last to be established, respectively. MC, CC, MGH, and JHH are located in the US and UNH is located in Canada. UHN is a public hospital, and the other four are private hospitals. All five hospitals are registered as nonprofit organizations.

**Table 1:** The general information of the world’s top five ranked hospitals

Hospitals	Mayo Clinic - Rochester	Cleveland Clinic	Massachusetts General Hospital	Toronto General Hospital - University Health Network	The Johns Hopkins Hospital
Ranking 2021	1	2	3	4	5
Country	USA	USA	USA	Canada	USA
Since	1864	1921	1811	1812	1889
Care System	Private	Private	Private	Public	Private
Sector	NFP	NFP	NFP	NFP	NFP
University/Affiliated	Mayo Clinic School of Medicine	Cleveland Clinic Lerner College of Medicine	Harvard University Affiliated	University of Toronto	Johns Hopkins University

**Note**: NFP (nonprofit hospital) A nonprofit hospital does not make a profit for the hospital owner from the funds collected for patient services

## Results

### Theme 1: Five hospitals’ descriptions of their missions

Table 2 shows the missions of the five hospitals [31–36]. We reviewed the missions of the five hospitals as described in their documents, and from the text, we found that MC focuses on inspiring hope and promoting health; CC focuses on caring, research, and education; MGH emphasizes delivering the very best health care, advance the care and to improve the health and well-being of the diverse communities; UHN refers to transforming lives and communities, and JHH desires to improve the health of our community and the world.

**Table 2:** The mission of the world’s top five ranked hospitals

Mayo Clinic - Rochester	Cleveland Clinic	Massachusetts General Hospital	Toronto General Hospital - University Health Network	The Johns Hopkins Hospital
Inspiring hope and promoting health through integrated clinical practice, education and research.	Caring for life, researching for health, educating those who serve.	Guided by the needs of our patients and their families, we aim to deliver the very best health care in a safe, compassionate environment; to advance that care through innovative research and education; and to improve the health and well-being of the diverse communities we serve.	Transforming lives and communities through excellence in care, discovery and learning	To improve the health of our community and the world by setting the standard of excellence in patient care.

### Theme 2: Five hospitals’ descriptions of their vision

The vision information of the five hospitals is collected and presented in Table 3. In the MC vision, the hospital’s goal is to transform medicine to connect and heal, while the CC vision is to be the best place for care and the best place to work in healthcare. The vision of Center for Community Health Improvement (CCHI) [36], a division of MGH, emphasizes the desire for a healthy, safe, and prosperous community and focuses on addressing health issues through the pursuit of equity, while the vision of UHN is to create a healthier world. Finally, JHH’s vision is to push the boundaries of discovery, transform health care, advance medical education, and create hope for humanity. Together, JHH’s vision will deliver the promise of medicine.

**Table 3:** The vision of the world’s top five ranked hospitals

Mayo Clinic - Rochester	Cleveland Clinic	Massachusetts General Hospital	Toronto General Hospital -University Health Network	The Johns Hopkins Hospital
Transforming medicine to connect and cure as the global authority in the care of serious or complex disease.	To be the best place for care anywhere and the best place to work in healthcare.	At CCHI, we envision healthy, safe and thriving communities where all people have equitable access to employment, food, education, housing and a high-quality health care system that addresses these and other social determinants of health.	A Healthier World for all.	To pushes the boundaries of discovery, transforms health care, advances medical education and creates hope for humanity. Together, we will deliver the promise of medicine.

**Note**: An extensive search failed to find the MGH vision, so the vision of the Center for Community Health Improvement (CCHI), a division of MGH, was used to replace this section (This result may not represent the entire MGH but can serve as a reference.)

### Theme 3: Five hospitals’ descriptions of their value

Table 4 illustrates the five hospitals’ practices in achieving their values, i.e., Mayo clinic’s values are that the needs of the patient come first. Mayo’s physicians and Franciscan Sisters define the values as putting the patient’s needs first, and they continue to guide MC’s operations to this day. MC’s values are also explained in the hospital’s values guidebook [37]: they treat everyone in MC’s diverse community, including patients, their families, and co-workers, with respect. Adhere to the highest standards of professionalism, ethics, and personal responsibility to live up to the trust patients place in the hospital. Provide the best possible care, treating patients and families with sensitivity and compassion. Inspire hope and foster a sense of well-being for the whole person, respecting physical, emotional, and spiritual needs. Value the contributions of all, blending the skills of individual staff members in an unparalleled collaboration. To energize the organization and improve the lives of those we serve through each staff member’s creativity and unique talents. Deliver the best results and highest quality of service through the efforts of each team member. Finally, sustain and reinvest in MC’s mission and extended community through the wise management of human, natural, and physical resources.

**Table 4:** The value of the world’s top five ranked hospitals

Mayo Clinic - Rochester	Cleveland Clinic	Massachusetts General Hospital	Toronto General Hospital - University Health Network	The Johns Hopkins Hospital
The needs of the patient come first	Quality & Safety Empathy Inclusion Integrity Teamwork Innovation	Excellence in patient care every day. Outstanding service to our patients, their families and our referring healthcare providers. Access to clinical care for all patients at the right time, in the right place by the right clinician. Seamless integration of research and clinical care for the benefit of our patients. Basic research that advances science and increases our understanding of the nervous system and its functions. Efficient and rapid translation of laboratory advances to patient care. Teamwork and collaboration. Education and mentorship for all our trainees and staff.	Safety, Compassion, Teamwork, Integrity, Stewardship.	Excellence & Discovery Leadership & Integrity Diversity & Inclusion Respect & Collegiality

CC’s values are stated in a few words [32], where “Quality & Safety” represents CC’s efforts to ensure the highest standards and superior results through effective interactions, decisions, and actions. “Empathy” means CC’s efforts to imagine what another person is going through, to try to alleviate suffering, and to create as much joy as possible. “Inclusion” represents the hospital’s intention to create a compassionate environment of belonging where all people are valued and respected. “Integrity” in the values is the adherence to high ethical principles and professional standards and a commitment to honesty, confidentiality, trust, respect, and transparency. “Teamwork” in the values is illustrated by CC’s commitment to working together to ensure the best possible care, safety, and well-being for patients and other caregivers.

MGH’s values suggest providing excellent care and excellence to patients and enhancing research and teamwork [33], followed by innovation, which refers to hospitals driving small and significant changes to transform health care. UHN’s values, on the other hand, are based on the five areas of safety, compassion, teamwork, integrity, and stewardship to achieve the mission and vision of the hospital [34]. “Safety” provides the safest possible care for patients and staff. “Compassion” is about kindness and respect in every interaction. “Teamwork” is about collaboration, cooperation, and diversity, and “Integrity” is about ethics, respect, and responsibility. Finally, “Stewardship” is about optimizing UHN’s resources for the greater public good.

Similar to CC and UHN, JHH’s values are demonstrated by a few keywords, “Excellence and Discovery,“ which means being the best. JHH is committed to excellence in quality and service by encouraging curiosity, seeking information, and creating innovative solutions. “Leadership and Integrity” is about being a role model, inspiring others to be their best, and having the courage to do the right thing. “Diversity and Inclusion is about being open and embracing and valuing diverse backgrounds, opinions, and experiences, while “Respect & Collegiality” is about being compassionate, listening to understanding, and accepting the unique skills and knowledge of others.

## Discussion

It was also clear that all five hospitals had a clear mission, vision and values, regardless of whether they were from the private or public sector or what management model they were implementing. In order to achieve its objectives, each hospital includes in its conceptual matrix evidence of an MVV, which is consistent with the findings indicated in previous studies that hospitals should be open to the public about the MVV that constitutes the code of ethics, as it is a subject with ethical, legal and social responsibilities [38]. Between MVV, much of the content of the five hospitals is consistent and sometimes overlaps. All contents address quality of care, clinical areas, and technical and organizational management, valuing human dignity and focusing on an ethical paradigm of individuality, responsibility, and care. Healthcare delivery outcomes are shaped by considering the MVV defined by healthcare organizations as a model to guide them to practice and obtain excellence in performance.

### Mission

#### Establishing the concept of the quality and excellence of health in the mission

The five hospitals’ mission statements mention quality and excellence, education and research, community engagement, access and equity, sustainability, belonging, and innovation. The missions of all five hospitals are related to life health and health care and serve a range of populations from individuals to communities to the world. As demonstrated in the results, three hospitals have inserted the pursuit of health quality and excellence into their missions. Research indicated that nearly all hospitals have adopted a management excellence approach and set goals within this framework to improve the performance of their health services organizations [39, 40].

Meanwhile, the missions of the five hospitals show different levels of emphasis on clinical, research, and teaching, with MC focusing on all three, CC focusing on research and education, MGH and JHU on community health, and JHHs on standards of care. Four hospitals mentioned education and research in their missions. Although JHH does not mention research and education in its mission, its parent institution, Johns Hopkins Medicine (JHM), does mention research and education at length in its mission [41]. After the day of the first COVID-19 case, the primary source of global data was reported by Johns Hopkins University in the United States. The first global real-time coronavirus surveillance system was launched by the Johns Hopkins University Center for Systems Science and Engineering (JHU CSSE): Coronavirus Resource Center [42]. As of June 1, 2022, the dashboard has served a global audience for over 30 months, totaling over 226 billion feature layer requests and 36 billion page views [42]. Therefore, the academic health center, such as JHH, MGH, and UHN, can share common resources with the university, not only for the university’s research but also in other fields (such as technology, law, engineering, etc.).

#### Describe the clinical, research and educational positioning in the mission according to their circumstances

The role of university-affiliated hospitals has traditionally been defined by its “tripartite” mission: education, research, and clinical care [43]. These three missions have become ends in themselves rather than activities that support a common goal. Research cannot and should not become a mission in and of itself, nor should education or clinical care. All three mission areas must act together to advance a common goal: a healthy future for all. In 2021, the Association of American Medical Colleges (AAMC) authors proposed a new framework that builds on the three missions described above and expands on the fourth mission, community collaboration. The AAMC’s call to prioritize community collaboration and health equity as pillars of the academic medicine mission [44]. This new mission may bring new inspiration to the world’s hospitals in the post-Covid-19 epidemic era.

All five hospitals are affiliated or partnered with medical schools in this study. Hospitals affiliated with universities usually combine teaching and education with saving lives, clinical, research, and talent training, allowing students to learn in practice and doctors to conduct clinical medicine and research work. In managing hospitals, hospitals affiliated with universities are usually larger, more resourceful, and have more substantial research capabilities. In addition, teaching hospitals are often considered to promote higher quality care, including treating rare diseases and complex patients, providing specialized services and advanced technologies, and conducting biomedical research [45].

It can be seen that the mission of evolving excellence in healthcare services, research, and education can embody the quality of healthcare services in the future of hospital management.

### Vision

#### Add a vision for individuals, families, communities and the world based on the location and positioning of the hospital

In our study, all five hospitals have a global vision, aiming for the health and well-being of all people with inclusive values, humanitarian-based health care, and health services. Moreover, all five hospitals highly emphasize collaboration and leadership with their colleague teams.

After comparing the visions of the five hospitals, we found that MGH and JHH are from the individual, the family, and the community to the global. In other words, their visions are covered from the microscopic to the macroscopic. MC, UNH, and CC mentioned changing the lives of their communities, and in parallel to clinical care, they also focus on the integration of medical education, health research, and health services.

#### In accordance with the evolution of society, equity is starting to become an important part of the vision of a good hospital

We found that MGH included health equity in its vision, while the other four hospitals did not clearly state this in their vision. The World Health Organization defines health inequity as a systematic difference in the health status of different populations, which has been a worldwide concern for many years. Health inequality is a multisectoral problem that significantly impacts people and communities (health, society, economy, etc.) [45–47].Previous studies have shown that medical professionals are biased against specific populations, which hinders their ability to provide adequate care [48]. Therefore, to avoid the prejudice of these medical professionals, MGH may seriously affect the medical quality results (for example, compliance with medical recommendations, cancer screening recommendations, and drug treatment plans). In the MGH vision, the maintenance of community health equity is particularly emphasized to reduce health inequity [49].

#### The emphasis on staff education and training in the vision is one of the most important factors in improving the quality of care

Finally, CC and MGH mentioned the training of talents and the employees’ working environment. Unfortunately, many managers and owners rarely consider employee education. They do not think it is part of their responsibility. If they do, they have no time to do it. The same situation exists in hospitals. A previous study indicated that doctors might believe their energy, education, and training should focus on patient care and research. Although doctors play a vital role in staff development, staff education should be the responsibility of hospital managers [47]. However, the training of employees and doctors will be rewarded in many aspects, such as leadership, effective communication, team development, and conflict resolution training, all of which can significantly improve performance [50]. Through adequate education and training, the hospital can be guided to declare its vision and realize its goals based on a clear mission, vision, and values. It can provide patients with high-quality health services, winning the recognition of patients and their families.

### Values

#### Incorporating patient needs and patient-centeredness into values can enhance effective communication, protect patients’ rights and improve the quality of care

Different from the values of MGH, JHH, UNH, and CC, only Mayo Hospital takes the needs of patients as a core value. MGH, JHH, UNH, and CC all emphasized the importance of hospital management in realizing the hospital’s mission, vision, and value and explained the importance of hospital management from different perspectives. The values of the above four hospitals all reflect quality and excellence, safety, integrity, and teamwork. For example, in the documents of Cleveland Hospital, we can see that the quality and safety of care, the compassion and integrity of patients, and the teamwork and innovation in the hospital are respectively included in the value description. The values of MGH, UNH, and JHH have similar descriptions.

Mayo Hospital has stated its value to put only the patient’s needs first, reflecting the current trend: “patient-centeredness. Patient-centered care is globally recognized as high-quality and high-value healthcare that emphasizes extensive patient and family involvement in health-related decision-making and healthcare services that meet patients’ needs, preferences, and values [51]. The mission of ‘inspiring hope and promoting health’ and the vision ‘transform medicine to connect and heal’ of MC has been more reflective of the patient-centered theme of hospital culture at MC. When patient-centered, medical and nursing staff can view from the patient’s perspective, enhance effective communication, and thus protect patients’ rights and improve the quality of medical services [52].

In summary, we found that these five hospitals’ MVVs were closely related to the overall management of the hospitals, which broadly included hospital safety, integrity, and teamwork. Value is for mission and vision achievement. While all the core values were related to performance excellence, there were varying degrees of difficulty in implementation among the core values. However, not all core values are equally easy to achieve. It means that some core values are more accessible to practice than others and can be used as benchmarks for hospitals with low overall performance excellence. For example, the Baldrige National Quality Award has all of the core values presented in it that are related to performance excellence. Projects with “entry-level” core values, such as patient-centered excellence, social responsibility, and community health, are easier to achieve than innovation management or agile management [53]. As far as hospital values are concerned, the top five hospitals have shared values. At the same time, it is imperative to emphasize safety, integrity, workforce management, and patient-centered values, which can help hospitals establish benchmarks and formulate strategies.

### Strengths and limitations

This study has two advantages. First, we selected the top five hospitals with the highest reputation in the world, analyzed what the five hospitals have in common in MVV, and examined the common elements of these high-quality hospitals in MVV so that hospital managers can learn their common characteristics. Second, we discovered the heterogeneity of five hospitals in MVV and found the individual high-quality elements of each hospital in its MVV, so that hospital managers can think deeply and apply this new knowledge to the advantage of their own hospital’s MVV.

Some limitations in our study should be noted. First, we used secondary sources currently publicly available on the Internet. These data are individually disclosed to the public of each hospital, such as terminologies, formats, and other information that may not necessarily have a consistent description. Second, given the accuracy of information comprehension, we finally included five hospitals in North America to obtain the most publicly available information about the MVV in English. In addition to this, we considered in our study that there may also be differences in MVV between public and private hospitals. However, of the top five hospitals, only UHN is a public hospital and is not in the same country as the other four, so more public hospitals will be included for comparison in future studies. Finally, although we collected various publicly available documents and website materials to analyze and compare MVV across hospitals and eventually found that MVV in hospitals of excellence had standard features, we recommend further research using site visits in the future to receive more in-depth insights into the content of MVV. In addition to MVV, leadership, patient relationship management, quality management, strategic management, performance management and social good in these five hospitals are also worthy parts of study for hospital managers. We will discuss each element in detail in other articles and will compare the inclusion of hospitals that are lower down the rankings, or even not on the list.

## Conclusion

The development of a mission is a good reflection of the focus and direction of the hospital’s leaders and leadership, as well as the hospital’s unique history, the hospital’s role in the community, social context, human health orientation, and expectations of the part of healthcare providers, showing more about social responsibility and sustainability. Furthermore, in the vision of the five hospitals, there is a consensus on the vision of the hospital with a focus on: the equity of patients and staff, the development of staff and even staff leadership, which can help the hospital attract top staff and improve the efficiency and results of hospital management to ensure patient service. Finally, in the development of values, all five hospitals have a framework of guiding principles for achieving performance standards such as patient-centeredness and improving quality of care, which can significantly help hospitals to develop strategic plans and improve their daily management with values as a benchmark.

## Data Availability

All data and materials generated or analysed during this study are included in this published article.

## Abbreviations

MVV: Mission, Vision, Value
MC: Mayo Clinic
CC: Cleveland Clinic
MGH: Massachusetts General Hospital
UHN: Toronto General Hospital – University Health Network
JHH: Johns Hopkins Hospital
CCHI: Center for Community Health Improvement
JHM: Johns Hopkins Medicine
